# An Attempt to Establish a Common Animal Model for Hepatorenal Fibrosis in Rats

**DOI:** 10.1155/2017/8260508

**Published:** 2017-08-01

**Authors:** Manoj Hang Limbu, Liu Lei, Cheng Zhengyuan, Liu Jing, Zhang Xiaoyi, Chen Pingsheng

**Affiliations:** Department of Pathology and Pathophysiology, Medical College of Southeast University, Ding Jia Qiao, Nanjing 210009, China

## Abstract

It is already a proven fact that there exists a relationship between CLD (chronic liver disease) and kidney disease but still there is no available combined animal model of liver and kidney fibrosis on the same animal. An animal model is one of the important research tools in the field of medical science because it is important to build a model that can simulate the disease condition so that the particular disease can be studied. Therefore, the aim of this study is to build a less expensive, less time consuming, and reproducible model of hepatorenal fibrosis on rats. We administered combined intraperitoneal injection of CCl_4_ (Carbon Tetrachloride) and BSA (Bovine Serum Albumin) on a female Wistar rats. At the end, the liver and kidney tissues were examined under microscope to see whether we were successful in establishing the model or not. The results show that liver fibrosis was marked but the changes on the kidneys were mild. In this study, we were able to induce significant fibrosis in the liver and early stages of fibrosis in the kidneys. The result also demonstrated that the addition of BSA conferred a liver protective effect against CCl_4_ induced hepatotoxicity, whereas combination of CCl_4_ and BSA proved to be detrimental for kidneys.

## 1. Introduction

There are many diseases in which there is a coexistence of both liver fibrosis and kidney fibrosis [1, 2], but currently available animal models are only of either liver fibrosis or kidney fibrosis alone. There are no common combined animal model of liver fibrosis and kidney fibrosis. Since extensive organ fibrosis is the hallmark of both chronic liver and kidney diseases regardless of their etiology, therefore, preventing or reversing fibrosis is still one of the main strategies in managing these diseases. The current treatment strategies for combined liver-kidney fibrosis are not effective [3, 4] and there is a need for new treatment strategies. For this purpose, there is a need for an effective common animal model of hepatorenal fibrosis for conducting further research. An animal model has been crucial for the study of diseases in the past and present. Despite the shortcomings of animal models, it will continue to be one of the important research tools in the field of medicine [5]. In this study, we attempted to establish a common model of hepatorenal fibrosis in rats which would represent a disease in which both liver and kidneys are fibrotic. This model would serve as a medium for further study in the field of hepatorenal fibrosis and aid in the discovery of new therapeutic targets.

We used combined intraperitoneal injection of Carbon Tetrachloride (CCl_4_) and Bovine Serum Albumin (BSA) on rats. CCl_4_ is known to be both hepatotoxic and nephrotoxic. There is a high success rate of inducing liver fibrosis with the CCl_4_ injections alone, but its nephrotoxic property is not severe enough to induce kidney fibrosis, whereas BSA is not hepatotoxic in an unsensitized rat but it has been used to simulate proteinuria of chronic kidney disease in a partially uninephrectomized rats. The requirement of decent surgical skill and equipment for performing partial nephrectomy leads to increased cost and extended duration of the study. Therefore, the aim of this study is to design a common hepatorenal fibrosis rat model using both CCl_4_ and BSA to eliminate the need of a surgical procedure and subsequently save time and cost along with significant induction of fibrosis in both liver and kidney.

## 2. Materials and Methods

### 2.1. Animals

Twenty-five female Wistar rats of age of 6 weeks, each weighing around 200 ± 25 gram, were purchased from Laboratory Animal Centre of Yangzhou University, Yangzhou China. The animals were housed at Animal Experiment Centre of Medical College of Southeast University, Nanjing, China. Animals were housed 5 per cage in a controlled environment of temperature (22°C) and humidity (55 ± 5%) with a 12-12-hour light-dark cycle. Tap water and standard rat pellets were given ad libitum. Animals were killed at the end of the 10th week via Sodium Pentobarbital injection and subsequent cervical dislocation. The study protocol was approved by the ethics review committee for animal experimentation of Southeast University.

### 2.2. Study Protocol

All animals were allowed to acclimatize for one week and then were randomly divided into the following four groups: Model group with 10 rats, BSA control group with 5 rats, CCl_4_ control group with 5 rats, and Normal control group with 5 rats. Each rat from model group received 1.5 mL/kg of 30% CCl_4_ in olive oil along with 1 gm of BSA as a 3 mL solution in NS (Normal Saline), BSA control group only received 1 gm of BSA as a 3 mL solution in NS, CCl_4_ control group only received 1.5 mL/kg of 30% CCl_4_ in olive oil, and Normal control group received nothing. CCl_4_ was injected intraperitoneally twice a week for 10 weeks. BSA was injected intraperitoneally 7 days a week for 8 weeks and then five days a week through week 9 and week 10. Animals were weighed and recorded every two weeks.

### 2.3. Light Microscopy

Livers and kidneys were dissected, weighed, and fixed in a 10% neutral buffered formalin. Paraffin sections were prepared from both liver and kidney tissues and stained with HE (Hematoxylin and Eosin) stain and Picro-Sirius red stain. Quantification of fibrous tissue deposition was done on Picro-Sirius red stained sections via image-j software; 10 random low power field images were selected and the mean area of collagen deposition was expressed as percentage.

### 2.4. Immunostaining

Immunohistochemistry for *α*-SMA (*α*-smooth muscle actin) was performed on paraffin embedded sections. Sections were first deparaffinized in a serially graded dilution of xylene; they were then rehydrated by washing in a graded dilution of ethanol and finally washed in distilled water. Next, heat induced antigen retrieval technique was performed followed by blocking of endogenous peroxide using 3% H_2_O_2_. The sections were incubated with a primary monoclonal anti-*α*-SMA antibody (Booster company) overnight at 4 degrees Celsius. In the next step, unbound areas were blocked using blocking polymer and the sections were incubated with a secondary antibody (Booster company) for 30 minutes at a room temperature. Finally, visualization with DAB kit (Booster company) was done and assessed under microscope.

Standard indirect immunofluorescence technique as described previously was used for staining of IgG, IgM, IgA, C3, and C1q on a frozen section of renal tissues; two sections each from CCl_4_ group, BSA group, and Model group were studied [6].

### 2.5. Electron Microscopy

Renal cortex samples, two from each group, were fixed in a 2.5% glutaraldehyde and sent to electron microscopy department for the preparations of ultrathin sections. The tissues were first washed and postfixed in a 1% osmium tetroxide and then were dehydrated in a series of ethanol and propylene oxide. The tissues were then embedded in an epoxy resin. Ultrathin sections were cut and mounted on a grid and the grid was stained with a uranyl acetate and lead acetate and finally observed under transmission electron microscope.

### 2.6. Biochemical Analysis

Blood samples were collected from direct heart puncture and serum was prepared. Total serum protein, serum albumin, serum globulin, ALT (Alanine transaminase), AST (Aspartate transaminase), BUN (Blood Urea Nitrogen), and creatinine were measured using the automatic analyzer.

Twenty-four-hour urine was collected via metabolic cage a day before killing and the volume was measured and recorded. The total urinary protein was estimated using a ready to use Bradford Assay (Nanjing Jiancheng Co.).

### 2.7. Statistical Analysis

Kruskal-Wallis test analysis was done using SPSS software; value of equal to or less than 0.05 was considered significant and a modified *P* value of equal to or less than 0.0083 was used for pairwise comparison.

## 3. Results

### 3.1. Physical Parameter

**Figure 1:**
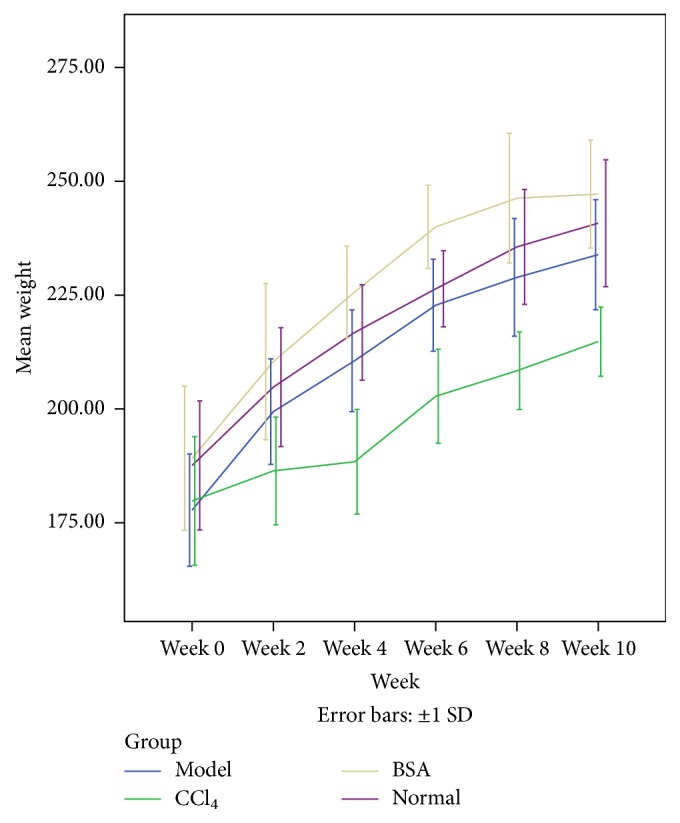
Graphical presentation of weight changes among different groups at different weeks throughout the duration of study. Weight gain is highest in the BSA group and lowest in the CCl_4_ group.

All the rats from the Normal group, BSA group, and Model group except for two rats in the Model group appeared to be healthy and active throughout the duration of the study. Rats from CCl_4_ group looked sick starting from the second week of the study until the time of killing. The mortality rate due to overdose and other circumstances were zero. Body weight from all the groups showed increasing tendency but the weight gain was highest in the BSA group followed by Model group and then Normal group. CCl_4_ group showed the lowest weight gain (Figure 1).

### 3.2. Histopathology

Grossly, livers from the model group were enlarged and pale looking; the borders were blunted and had a coarse surface. Livers from the CCl_4_ group were smaller compared to other groups and looked pale with a coarse surface, whereas livers from BSA group and the Normal group did not show any change. Kidneys from all the groups showed no visible differences. The mean and the mean percentage of the organs are summarized in Table 1.

**Figure 2:**
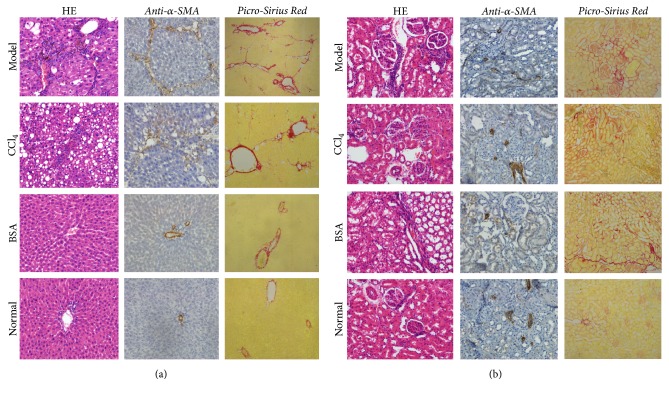
Comparison of histopathology among the groups. (a) Liver histopathology shows marked liver fibrosis in the model group and CCl_4_ group. HE stain in the Model group shows periportal fibrosis and fibrous septa formation. Anti-*α*-SMA antibody positive staining is observed in the fibrotic areas and periportal/perivascular areas. Picro-Sirius red staining corresponds to the changes seen in HE stain. Histopathology of CCl_4_ group also shows similar changes as that of Model group along with fatty changes. BSA group and Normal group show no changes except for edema and mild connective tissue proliferation in the BSA group. (b) Histopathology of kidney from Model group and BSA group shows focal areas of connective tissue proliferation and inflammatory cell infiltrates. Anti-*α*-SMA antibody positive staining is observed in the fibrotic regions and perivascular regions of Model, CCl_4_, and BSA group. Picro-Sirius red staining shows similar intensity of collagen staining between Model, CCl_4_, and BSA group.

Histologically, ten out of ten livers in the Model group showed distorted normal architecture varying from mild to moderate degree and multiple patchy areas with incomplete fibrous band formation. No fibrotic nodules were seen but there was a marked cellular swelling and inflammatory cell infiltrates. Fatty degeneration in Model group were less prominent than in the CCl_4_ group. Five out of five livers from CCl_4_ group also showed similar changes as with model group except for the higher degree of fatty change than in the Model group. BSA group also showed mild proliferation of connective tissue around the portal and vascular region. Livers from Normal group showed no abnormality (Figure 2(a)). In Model group, the kidneys showed the focal areas of connective tissue proliferation around the collecting ducts along with the occasional areas of protein cast. Lymphocytic infiltrations in the perivascular region and peritubular region were also seen. The similar changes were observed in the kidneys of CCl_4_ group and BSA group but to a lesser degree. The Normal group showed no marked abnormalities (Figure 2(b)).

**Figure 3:**
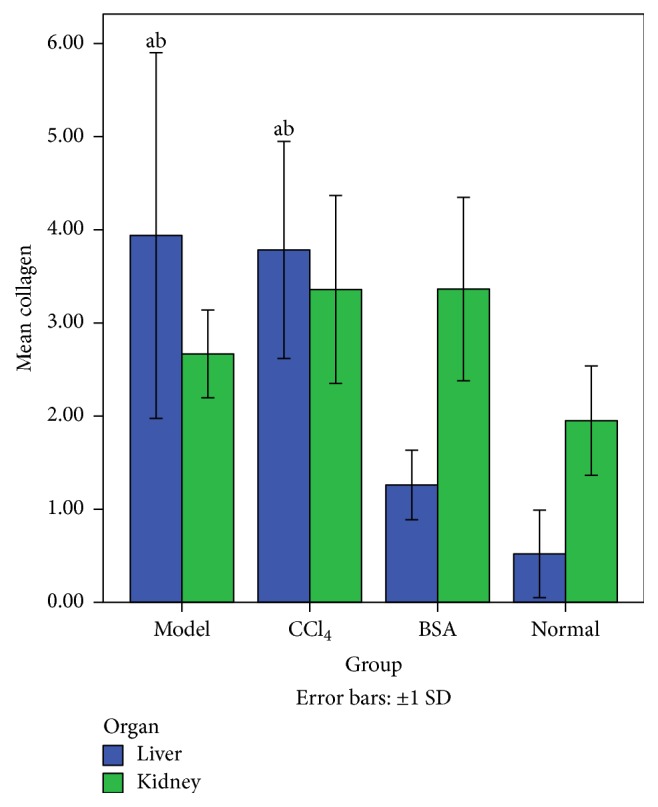
Graphical presentation of collagen deposition among various groups. The amount of collagen deposition in the livers of Model group and CCl_4_ group is significantly higher than that of other groups but difference is insignificant in comparison to each other. Collagen deposition in the kidneys is insignificant (*Note*. a: significant in comparison to Normal group, b: significant in comparison to BSA group).

Quantification of collagen deposition as estimated by image-j software showed that the collagen deposition in the liver was highest in the Model group (insignificant in comparison to CCl_4_ group). In the kidneys, the differences in the collagen content were insignificant (Figure 3).

### 3.3. Immunostaining

*α*-SMA antibody positive cells in the livers of Model group were present in the fibrotic areas, perivascular areas, and periportal areas and represented about 1/3rd of the total area. CCl_4_ group also showed a similar trend as that of Model group. The Normal group were positive only around the periportal areas. The *α*-SMA antibody positive cells in the kidneys of Model group, CCl_4_ group, and BSA group were strongly positive around perivascular regions, in the areas of connective tissue proliferation, and in areas of interstitial tissues. The above changes were more pronounced in the kidneys of the Model group. The kidneys from the Normal group showed strong positivity around perivascular regions only (Figure 2).

**Figure 4:**
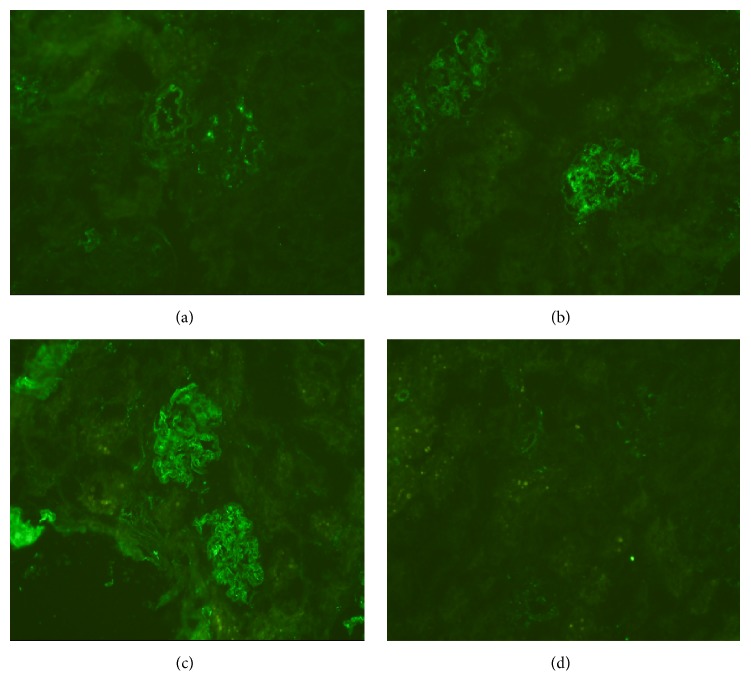
Immunofluorescent microscopy of renal tissues. (a) CCl_4_ group shows granular deposits of IgM in the mesangium. (b) BSA group shows granular deposits of IgM in the mesangiocapillary region. (c) Model group shows granular deposits of IgM in the mesangiocapillary region. (d) Model group shows granular deposits of IgG in the mesangium.

Immunofluorescence study showed granular deposition of IgM in the mesangiocapillary region of BSA group and Model group and in the mesangial region of CCl_4_ group. IgG was positive only in Model group around the mesangial region. IgA, C3, and C1q were negative in all groups that we studied (Figure 4).

### 3.4. Biochemistry

Serum protein level in the Model group was significantly higher than that of the CCl_4_ group and Normal group, but there was no difference in comparison to BSA group. There was no significant difference in the serum albumin level among all the groups. Serum globulin level also showed a similar trend as that of serum protein level.

**Figure 5:**
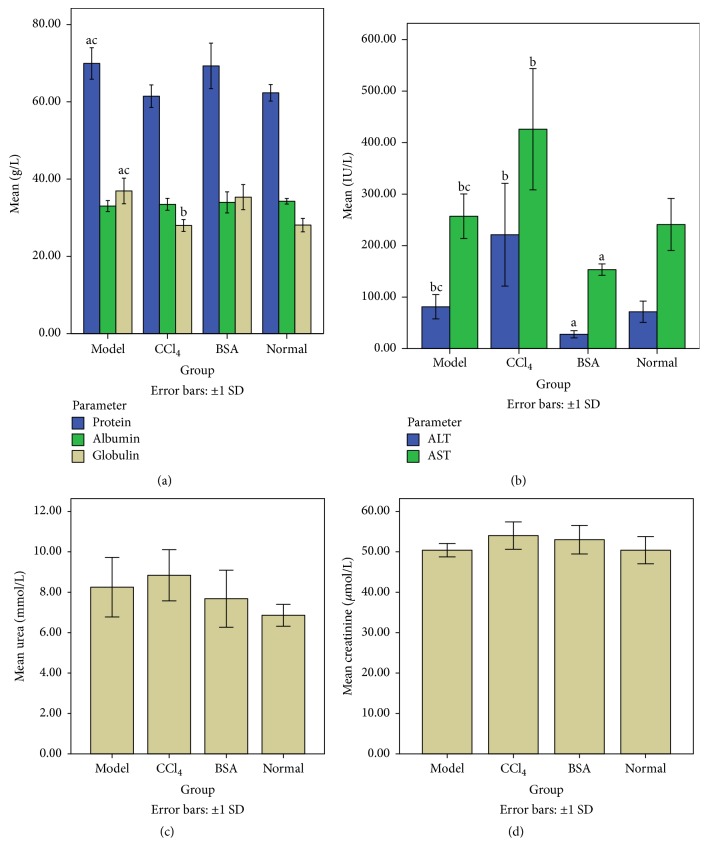
Graphical presentation of biochemical index of various groups. (a) There is no significant difference in the serum albumin levels among all the groups. (b) Serum ALT/AST shows similar trend among various groups. (c and d) Serum BUN and creatinine levels show no significant differences among the groups. (*Notes*. c: significant in comparison to* CCl*_*4*_ group, b: significant in comparison to BSA group, and a: significant in comparison to Normal group.)

Serum ALT level was highest in the CCl_4_ group (significant in comparison to BSA group, insignificant in comparison to Model group and Normal group) and lowest in the BSA group (significant in comparison to rest of the group). Serum AST level also showed a similar trend as that of serum ALT level. There was no significant difference in the level of serum BUN and serum creatinine among all the groups. The summary of the biochemical index is illustrated in Figure 5.

### 3.5. Urinary Protein

Twenty-four-hour urine output was highest in the Model group (significant in comparison to the Normal group but insignificant in comparison to CCl_4_ group and BSA group) and was lowest in the Normal group (significant in comparison to Model group and CCl_4_ group but insignificant in comparison to BSA group).

**Figure 6:**
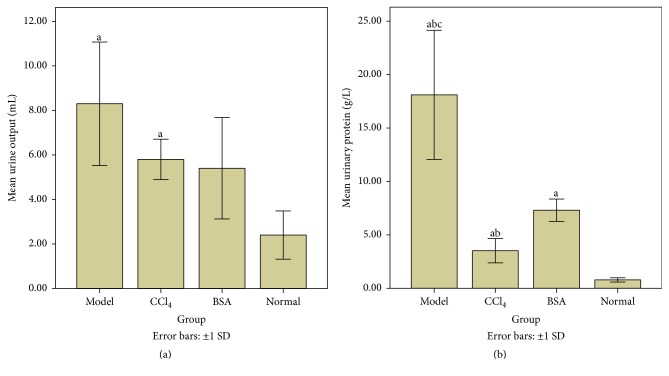
Graphical presentation of mean 24-hour urinary output (a) and mean proteinuria (b) in different groups. Model group shows significant increase in urine output as well as increased urinary protein in comparison to other groups. (*Note*. a: statistically significant in comparison to Normal group, b: statistically significant in comparison to BSA group, and c: statistically significant in comparison to CCl_4_ group).

Urinary protein as quantified by Bradford Assay showed that Model group had the highest amount of urinary protein excreted (significant in comparison to rest of the group). Urinary protein level in the Normal group was the lowest (significant in comparison to rest of the group). The results of urinalysis are summarized in Figure 6.

### 3.6. Electron Microscopy

**Figure 7:**
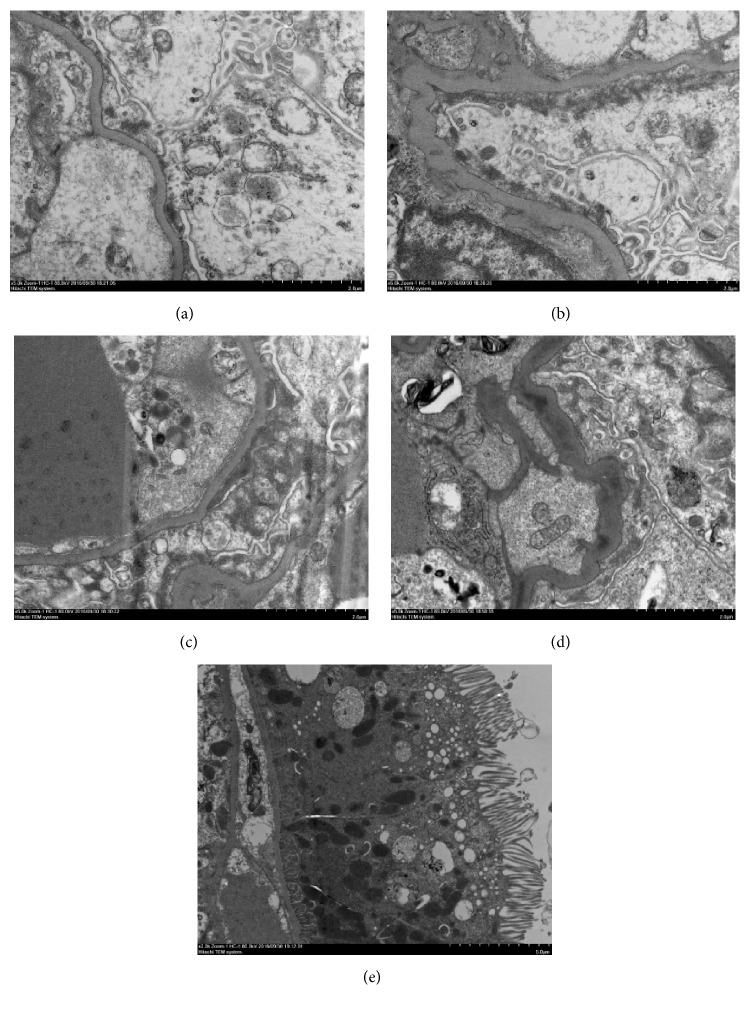
Electron microscopy of renal tissues. (a and b) BSA group shows subepithelial electron-dense deposits, GBM loss of trilaminar structure, podocyte swelling, and loss of foot processes. (c and d) Model group shows electron-dense deposits in the subepithelial as well as intramembranous region. (e) Proximal tubular cell of Model group shows senescent mitochondria, autophagosomes, and increased endocytosomes.

Due to the sampling error, glomerulus from the Normal group and CCl_4_ group could not be assessed. Glomerulus from both BSA group and Model group showed loss of trilaminar structure in the GBM along with podocytes swelling and loss of foot processes. Swollen mitochondria and increased endocytosomes could be seen in the podocytes of both groups. In the glomerulus of Model group, there were subepithelial as well as intramembranous deposition of electron-dense deposits, whereas in the BSA group electron-dense deposits were present in subepithelial region only. Ultrastructure of proximal tubular epithelium from the Model group revealed increased senescent mitochondria, autophagosomes, and endocytosomes (Figure 7).

## 4. Discussion

Renal involvement in the setting of liver disease is a fact and it is also a fact that there exists no reliable model of hepatorenal fibrosis up to date, which can simulate this condition in the real clinical settings. Therefore, sooner or later the initiative towards establishing a novel model of hepatorenal fibrosis must be taken.

The result of histopathology from this study showed that CCl_4_ can induce liver injury as well as renal injury, which were consistent with the findings of previous studies [7–9]. Histopathology from the BSA group did not show any hepatic lesions but only renal lesions; thus we can imply that BSA overload had no apparent effect on liver histology. Accordingly, we can also hint that renal lesions observed in the model group were because of combined effects of CCl_4_ toxicity and BSA overload. In this study, the hepatic lesions observed in Model group and CCl_4_ group were severe enough to produce a biochemical lesion, which correlated well with their respective histological lesions, whereas the renal lesions were not severe enough in any group to produce biochemical changes. However, these biochemical parameters are not the reliable markers of early fibrotic changes [10–12].

IgM positivity by immunofluorescence study was observed in all groups, whereas IgG positivity was only seen in the Model group. IgA deposition was not observed in any group. This finding differed from the previous studies demonstrating the glomerular deposition of IgA as an important feature of immune deposits in the kidneys of animal model and in the kidneys of human in kidney disease associated with chronic liver diseases [13–15]. But still this result demonstrated that immune complex deposition also occurred in this model. However, recent study suggests that it is the type and site of the immunoglobulins that are more important in determining the severity of renal disease [16–20]. Therefore, this study suggests that high level of urinary protein in the model group correlated with the deposition of both IgG and IgM in the mesangiocapillary region of its kidney. In contrast, BSA group showed IgM deposition in the mesangiocapillary region only while CCl_4_ group showed IgM deposition in the mesangial region only, both of which had lesser amount of urinary protein in comparison to Model group. These findings were further substantiated by the findings of electron microscopy, which demonstrated electron-dense deposits in the subepithelial as well as in the intramembranous region of the Model group, whereas, in the BSA group, the electron-dense deposits were observed only in the subepithelial region.

The possible mechanism of hepatorenal injury in this study could have been mediated by the generation of ROS (Reactive Oxygen Species) due to the direct effect of CCl_4_ on both liver and kidneys [21, 22]. The resulting oxidative stress in the liver and kidney from the effect of ROS generation could have led to liver fibrosis and renal epithelial/endothelial injury, respectively [1–3, 23]. Kidney might as well have been further affected by the generation of ROS due to the effect of subsequent liver fibrosis [24]. In addition to that, overloading of BSA might have aggravated the already mildly disrupted glomerular filtration barrier, which further aggravated the proteinuria. Past studies have also shown BSA overload alone is associated with the disruption of filtration barrier [25–27]. Therefore, we can also conclude that the cumulative effects of both CCl_4_ and BSA have worked in synchrony to aggravate the proteinuria. The proteinuria might have further invoked an immune response in the proximal tubular cells, which caused interstitial nephritis and finally led to fibrosis [28, 29]. The previous study on the pathogenesis of proteinuria associated renal injury also suggested that the disruption of the urinary protein homeostasis and subsequent increase in protein reabsorption in the proximal tubular cell might be the possible mechanism of renal injury [30, 31]. Electron microscopy of the renal sections from both BSA and Model group also showed disruption of filtration barrier, swelling of podocytes, and proximal tubular cells. Podocytes and tubular cells also showed increased senescent mitochondria, endosomes, and autophagosomes. Thus, the result of electron microscopy also supports the above reasoning.

The immunostainings of *α*-SMA in the fibrotic regions of all the groups were consistent with previous studies suggesting an involvement of myofibroblast in both organs. However, the origin of these mesenchymal cells has been disputed [32–35]. Many studies have proposed a various possible source of origin of these myofibroblasts in both organs while the EMT (Epithelial-Mesenchymal Transition) theory has been questioned [36, 37].

Overall the results in this study were not as expected from our preexperiment predictions. Prior to the beginning of this study, we conducted a preexperiment with similar protocol except that the dosage of CCl_4_ was 40% and that of BSA was 500 mg. The total duration was for 8 weeks. The histological hepatorenal lesions observed in that study were of higher degree than that of this formal experiment (results not published). Therefore, based on this we assume that the dosages of CCl_4_ are crucial in determining the extent of renal injury rather than the dosage of BSA alone. Accordingly, we suggest using higher concentration of CCl_4_ in the future to make an improvement to this model, even though high mortality rate might be of concern. Another striking finding we observed from the results of this study is that the BSA conferred a liver protective effect against the CCl_4_ induced toxicity which might have something to do with the antioxidative properties of albumin as discussed by other researchers [38]. On the other hand, the data clearly shows that combined administration of CCl_4_ and BSA is detrimental for renal functions.

Kidney disease in the setting of chronic liver disease manifests in various manners with distinct pathophysiology, and whether this animal model simulates the real clinical condition should be evaluated after further detailed investigations [39]. We are also aware that in this study the sample size was too small to draw a solid conclusion, but nevertheless we believe that we still achieved some success in an effort to establish a novel model of hepatorenal fibrosis. We hope that the findings from this study may serve as an important reference point for the study of similar type in the future.

## Figures and Tables

**Table 1:** Comparison of mean organ weight and mean percentage organ weight among various groups.

	Model	CCl_4_	BSA	Normal	*P*
Liver					
Average weight (gram)	9.824 ± 0.51^ac^	7.290 ± 0.24^b^	9.394 ± 0.45^a^	7.706 ± 0.45	*P* < 0.05
Weight/100 gram body weight	4.145 ± 0.22^ac^	3.302 ± 0.20^b^	3.910 ± 0.09^a^	3.153 ± 0.19	*P* < 0.05
Kidney					
Average weight (gram)	1.965 ± 0.10^ac^	1.522 ± 0.05^b^	1.953 ± 0.09^a^	1.587 ± 0.09	*P* < 0.05
Weight/100 gram body weight	0.833 ± 0.03^ac^	0.708 ± 0.02^ab^	0.792 ± 0.02^a^	0.645 ± 0.03	*P* < 0.05

*Notes*. ^a^Significant in comparison to Normal group; ^b^significant in comparison to BSA group; ^c^ significant in comparison to *CCl*_*4*_ group.

## Abbreviations

CLD: Chronic liver disease
CKD: Chronic kidney disease
CCl_4_: Carbon Tetrachloride
BSA: Bovine Serum Albumin
*α*-SMA: Alfa-smooth muscle actin
ALT: Alanine transaminase
AST: Aspartate transaminase
BUN: Blood Urea Nitrogen
NS: Normal Saline
ROS: Reactive Oxygen Species.
